# Association between inflammatory parameters and neuropsychiatric symptoms in COVID-19 patients treated in hospital del mar Post-COVID psychiatry functional unit

**DOI:** 10.1192/j.eurpsy.2023.878

**Published:** 2023-07-19

**Authors:** L. Vargas Puértolas, T. Legido Gil, E. Pechuán Martínez, À. Arroyo Núñez, S. Oller Canet

**Affiliations:** 1 Psychiatry; 2 Psychology; 3 Mental health nurse, Hospital del Mar, Barcelona, Spain

## Abstract

**Introduction:**

There is increasing interest in the association between neuropsychiatric symptoms in patients with COVID-19 and the proinflammatory status of the disease. Hospital del Mar Post-Covid Psychiatry Unit carried out a descriptive study to analyze the link between inflammation and mental health symptoms in COVID-19 patients.

**Objectives:**

Relate inflammation parameters to the presence of neuropsychiatric symptoms in COVID-19 patients treated at the Hospital del Mar Post-Covid Psychiatry Unit.

**Methods:**

A database of patients evaluated by the Post-covid Psychiatry unit was developed. Clinical variables, whether hospitalization is required and inflammation indicators during COVID-19 infection (PCR and IL-6 analytical values) were recorded.

Three screening scales for psychiatric symptoms were given to the patients: PHQ-9 for depression (1-4: minimal depression, 5-9: mild, 10-14: moderate, 15-19: moderately severe, 20-27: severe), GAD-7 for anxiety (cutoff point >=6) and PCL-5 for post-traumatic stress symptoms (cutoff point >6).

T-student statistics for independent samples and the pearson correlation were used to relate inflammation parameters to depression, anxiety and post-traumatic stress symptoms obtained from the scales.

**Results:**

149 patients were attended between may 2020 and april 2021. 78 patients had PCR value and 52 had IL-6 value. There is no correlation between the score obtained on the PHQ-9, GAD-7 and PCL-5 scales and PCR or IL-6 value.

There is no relation between being hospitalized for covid infection and the values of PHQ9 and GAD7. Patients hospitalized had lower scores in PCL-5 scale (t=2.67, p=0.009). There are no differences in the scale scores among patients requiring orotracheal intubation or not.

**Conclusions:**

In this descriptive study, inflammation parameters were not related with psychiatric symptoms in COVID-19 patients. Neither association was found between the inflammation parameters and the severity of COVID-19 symptoms, measured in terms of hospitalization requirement.

**Disclosure of Interest:**

None Declared

